# Occurrence and surgical repair of third degree perineal lacerations in adult female camels (*Camelus dromedarius*) by one-stage (Goetz) technique

**Published:** 2013-07-10

**Authors:** S. Anwar, G.N. Purohit

**Affiliations:** 1 *Al-Qattara Veterinary Hospital Abu Dhabi Food Control Authority, Al Ain, P.O.Box 1004, United Arab Emirates*; 2 *Department of Veterinary Gynecology and Obstetrics, College of Veterinary and Animal Sciences, Rajasthan, University of Veterinary and Animal Sciences, Bikaner, Rajasthan, 334001 India*

**Keywords:** Camels, One-stage repair, Perineal lacerations

## Abstract

Retrospective analysis of third degree perineal lacerations in 7 female camels (6-17 yrs of age) that were surgically corrected by one stage repair (Goetz technique) is presented. Majority (3/7) of the camels was primiparous and all parturitions had a history of calving assistance. Six (6/7) camels recovered by first intention of healing. Dehiscence of perineal structure occurred in only one camel due to infection and healed by second intention. Subsequent matings resulted in pregnancy in four camels and one camel died due to unrelated causes. We conclude that perineal lacerations can occur in primiparous camels with difficult assisted deliveries and that one stage repair of perineal lacerations in camels improves the perineal conformation and such camels may easily regain normal fertility.

## Introduction

Primiparous females are susceptible to injuries of the perineum, particularly during parturition (Dreyfuss *et al.*, 1990; Kazemi *et al.*, 2010). In cattle and mares, forced extraction of an oversized or maldisposed fetus from an insufficiently dilated birth canal predisposes them to perineal injuries in the form of lacerations (Straub and Fowler, 1961; Hudson, 1972; Colbern *et al.*, 1985; Dreyfuss *et al.*, 1990).

Perineal lacerations are much more common in mares compared to cattle and other domesticated species. The prominence of the vestibulo vaginal sphincter and remnants of the hymen in mares foaling for the first time are presumed to be responsible for most of these injuries (Kazemi *et al.*, 2010).

The powerful expulsive efforts and the rotation of the equine fetus from a dorso-ventral to a dorso-sacral position during parturition (Jeffcott and Rossdale, 1979; Frazer *et al.*, 1999; Purohit, 2011) renders the foals leg to exert undue pressure on the lateral and dorsal walls of the birth canal; thus increasing the chances of laceration (LeBlanc, 1999; Woodie, 2006). The foal’s hooves catch the dorsal transverse fold of the vestibulo vaginal junction, and the mare’s abdominal compression during foaling forces the foal’s foot into the roof of the vestibule (LeBlanc, 1999; Woodie, 2006).

Perineal lacerations are usually less frequent in camels (Tyagi and Singh, 1993; Tibary and Anouassi, 2000; Siddiqui and Telfah, 2010). Reports on the surgical corrections of perineal lacerations in camel are unavailable, although it has been mentioned that in camels with third degree lacerations matings should be carefully done to avoid the intromission of the penis into the rectum (Tibary and Anouassi, 2000). Reconstruction of third degree perineal lacerations appears necessary to quickly bring the female camels back to breeding soundness and for cosmetic reasons. The aim of this report is to describe the Goetz methodology of six bite suture pattern which we successfully used to repair third degree perineal lacerations in camels.

## Case selection

All female camels presented to the surgery section of veterinary hospital, Al Qattara, Al Ain, United Arab Emirates with third degree perineal lacerations from October 2009 to April 2011 were retrospectively reviewed. Only camels that had a tear through the rectovestibular septum, musculature of the rectum and vestibule, and the perineal body were included in this study and considered to have third degree perineal lacerations as described previously for mares (Aanes, 1988; Kazemi *et al.*, 2010).

**Fig. 1:**
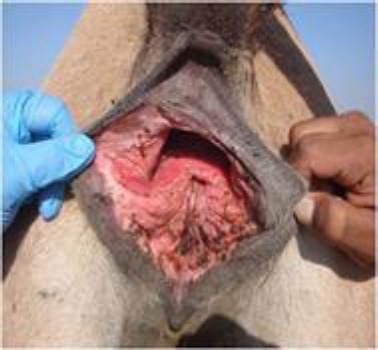
Third degree perineal laceration in a female camel.

Affected camels evidenced rectovaginal fistula (Fig. 1), constant or intermittent tenesmus and one camel had accumulation of urine in the torn vaginal vault. Since all the camels evidenced variable degree of inflammation, they were administered a combination of procaine penicillin, benzathine penicillin each 5000 IU/kg and dihydrostreptomycin sulfate 10 mg/kg(Inj. Penstrep 400 LA, Interchemie Holland) IM every 48 hours for 6 days and inj. Ainil (Ketoprofen, Invesa, Spain) at the rate of 2 mg/kg IV for 3 days. Some devitalized tissue was excised followed by application of diluted 7.5% povidone iodine solution over the wound surface.

## Surgical Technique

One month after the initial referral, camels were re-examined and once satisfactory resolution of the inflammation was obtained, surgery for the correction of perineal laceration was planned. Camels were kept off food for 5 days and water was withheld for one day prior to surgery to minimize fecal contamination during surgery.

**Fig. 2:**
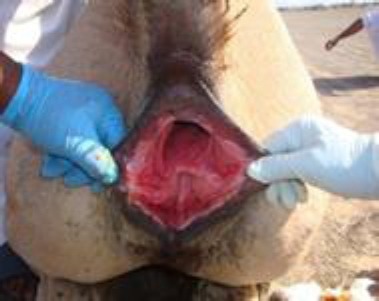
The surgical field prepared for surgery under epidural anaesthesia.

The animals were restrained in sternal recumbency and sedated by IV administration of 0.25 mg/kg of xylazine hydrochloride (XYL-M2, VMD, Belgium) along with epidural administration of 15 ml of 2% lignocaine hydrochloride (Lurocaine, Vetoquinol, France). The tail was tied to one side. Fecal material from the rectum was removed as far cranial as possible. The perineal region and vaginal vault were cleaned by repeated flushing with a mild povidone iodine solution according to standard surgical principles and were thoroughly dried (Fig. 2).

After preparation of the perineal region, Goetz technique (single-stage repair) described previously for horses (Belknap and Nickels, 1992; O’Rielly *et al.*, 1998; Phillips and Foerner, 1998) was used for suturing and closure of the lacerations.

**Fig. 3:**
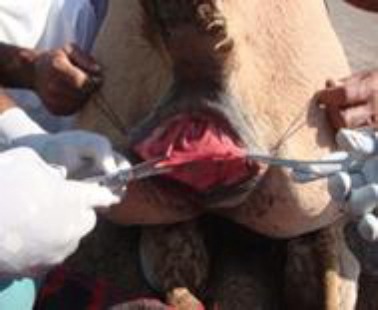
Commencement of dissection of vaginal mucosa.

The operative area was exposed by holding skin with Allis tissue forceps near the muco-cutaneous margin on each side of the disrupted dorsal commissure of the vulva (Fig. 3) and an incision was made along the scar tissue at the junction of the rectal and vaginal mucosa, separating the two structures.

**Fig. 4:**
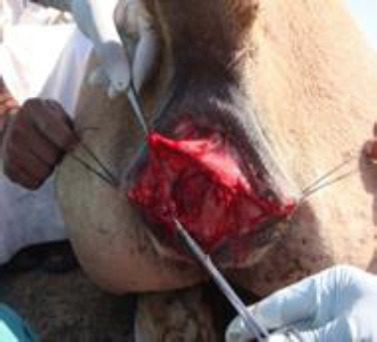
Vestibular and rectal tissue flaps are created by dissecting along the line of scar tissue.

After removing the scar tissue, the sub mucosa between the ventral aspect of the rectum and the dorsal aspect of the vagina was split cranially in a transverse plane for a distance of 3-5 cm. Further dissection was carried out caudolaterally along the right and left walls of the common vault of the rectum and vestibule, to the point of junction of labia at dorsal commissure of vulva (Fig. 4).

**Fig. 5:**
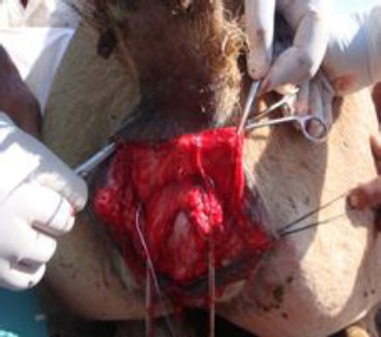
The rectovestibular septum was opposed with interrupted six bite suture patterns.

**Fig. 6:**
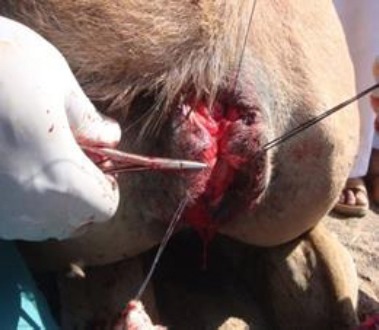
Perineum was constructed by simple interrupted sutures.

**Fig. 7:**
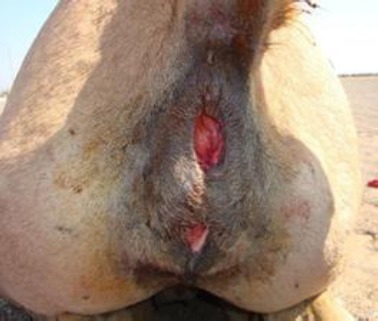
Closure of the skin of perineum by simple interrupted sutures.

Closure of recto vestibular shelf was accomplished by a six-bite suture pattern with No. 2 Polyglactin (Vicryl, Ethicon, USA) suture material, placed 0.5-1 cm apart starting just cranial to the defect and was terminated at the dorsal commissure of vulva (Fig. 5). The operation was completed by reconstruction of the perineum with single interrupted sutures using Polyglactin (Vicryl, Ethicon, USA) No. 2 in 2-3 layers commencing cranially and terminated caudally (Fig. 6). The skin of perineum was closed with simple interrupted Polymide suture (Dafilon, Braun, USA) USP No.1 (Fig. 7).

Postoperative care included daily cleaning of the suture line with 5% povidone iodine solution followed by spraying of nagasunt powder (Coumaphos: 3%, Propoxur: 2% Sulfanilamide: 5%, Bayer Germany). Injection Penstrep (penicillin-streptomicin, Norbrook UK) at the rate of 1ml/20 kg b. wt. was administered IM for 5 days and injection Ainil (Ketoprofen, Invesa Spain) at the rate of 2 mg/kg was administered IV for 3 days. Considering postoperative complications like atony of rectum, pain, constipation, tenesmus, and frequent attempt to defecation which may result in suture dehiscence; owners were advised to feed only a small quantity of green alfalfa hay every day throughout the postoperative period and if needed careful manual emptying of the rectum was advised. The skin sutures were removed approximately 3-5 weeks after surgery.

## Case Review

Records of history evidenced that majority (5/7) of the camels had delivered male calves at parturition when the injury occurred and only a small proportion (2/7) had delivered female calves (Table 1). Forceful extraction of the fetus was common to all affected camels. Camels delivering males were either primipara or in their second parity (Table 1). Camels were presented after variable times after the occurrence of the laceration (Table 1) and one camel had undergone surgery previously and was presented for the second time. All the female camels recovered uneventfully except one camel in which dehiscence of perineal sutures occurred following infection which healed by second intention.

**Table 1:** Case records of 7 female camels before and after repair of perineal lacerations.

S. No.	Age of dam (years)	Parity	Sex of fetus at time of injury	Time interval between parturition and surgery	Post-operative complications	Interval between repair and breeding	Results of breeding
1	17	6^th^	Female	3 years	None	1 month	Pregnant
2	7	1^st^	Male	9 months	Edema of perineum and vulvar lips	3 months	Barren
3	6	1^st^	Male	3 months	Dehiscence of perineal sutures	1 year	Pregnant
4	14	4^th^	Female	2.5 months	None	6 months	Pregnant
5	12	2^nd^	Male	2 years	None	2 months	Pregnant
6	9	1^st^	Male	1 year	None	None	Died due to unrelated causes
7	12	2^nd^	Male	3 months	None	6 months	Barren

Owners comfortably performed postoperative care including assisted evacuation of rectum. Follow-up carried out one month after surgery demonstrated no complications. Recto-vaginal fistula formation, urine accumulation, constipation and tenesmus were abolished and perineal conformation was improved by the surgical technique. Subsequent matings were carried out between 1 to 12 months following complete recovery and resulted in pregnancies in 4 out of total 7 camels (irrespective of the number of matings) during the first breeding season following surgery.

## Discussion

To the best of our knowledge, this is the first report on perineal lacerations in camels. Perineal lacerations in camels during the present study had a history of forceful extraction of fetus during a difficult parturition with majority of dams in their first or second parity and a large proportion of fetuses being male. It is considered that female dromedary camels carrying male fetuses have a slightly longer duration of pregnancy (Sharma and Vyas, 1971; Agarwal *et al.*, 1987) with the male calves being 3-5 kg heavier and larger sized at birth (Barhat and Chowdhary, 1980). It is thus possible that undue force was probably exerted during delivery of slightly larger fetuses from an insufficiently dilated birth canal which led to perineal lacerations. Similar origins of perineal lacerations in mares have been described previously (McKinnon and Vasey, 2007) and it has also been mentioned that primiparous mares and first-calf cows are by far the most commonly injured candidates with third degree perineal lacerations (Dreyfuss *et al.*, 1990; O’Rielly *et al.*, 1998; McKinnon and Vasey, 2007; Kazemi *et al.*, 2010) during parturition assistance.

In the present report four camels were aged above 12 years and possibly an older age could also be a risk factor for the occurrence of perineal lacerations.

Although different groups of authors slightly differ in their approach for repair of perineal lacerations in mares, the objective of all procedures is to rebuild the shelf of tissue between the rectum and the vestibule and to restore the structural integrity of the perineal body. During the present study third degree perineal lacerations in camels were successfully repaired by Goetz technique, a one-stage method described for mares and cattle (Dreyfuss *et al.*, 1990; Phillips and Foerner, 1998; LeBlanc, 1999; McKinnon and Vasey, 2007) and this technique offered improved fertility and conformational soundness in the perineal area.

6 out of 7 camels recovered by first intention of healing. Similar results were observed in previous studies on mares (Phillips and Foerner, 1998; Janicek, 2007; Ghamsari *et al.*, 2008). However, similar studies in camels are unavailable. In our study, one camel had dehiscence of perineal sutures and healing occurred by second intention. Similarly, single stage repairs of third grade perineal lacerations and recto-vestibular fistula in mares have revealed primary healing in majority of mares (Belknap and Nickels, 1992). Kazemi *et al.*, 2010 found no dehiscence of suture line in 7 treated mares.

It has been suggested that surgery should be delayed for 4-6 weeks because immediate repair of lacerations are generally unsuccessful due to edema and inflammation of lacerated tissue and the ensuing contraction of the muscles of the rectum and vestibule rapidly widen and lengthen the wound (Desjardins *et al.*, 1993; LeBlanc, 1999; Woodie, 2006). Such a delay permits epithelial re-growth to cover the damaged tissue. In the present work similar approach was followed although cases were presented after variables times subsequent to parturition and the occurrence of the problem (2.5 months to 3 years).

None of the camels showed signs of discomfort, pain or constipation during the recovery period. According to Ghamsari *et al*. (2008) the one stage repair is advisable for chronic lesions such as those treated in our study. In one of the camel in this study the surgical repair was attempted 2^nd^ time suggesting that the method can also be applied to aid the healing of chronic cases.

Previous studies in mares (Schumacher and Blanchard, 1992) have shown that endometritis could subside within 15 days after recto-vestibular repair and breeding could be allowed by artificial insemination. Studies also showed that subsequent fertility in mares and cows could be improved and 62.5 to 75 % of mares (Kasikci *et al.*, 2005; Kazemi *et al.*, 2010) and 71 % of cows (Dreyfuss *et al.*, 1990) became pregnant following one stage surgical repair. In the present report, four camels that had undergone surgery became pregnant, suggesting that one stage surgical repair can be efficiently employed in female camels with third degree perineal lacerations. The perineal conformation improved in all camels that underwent surgical repair.

We conclude that perineal lacerations can occur in primiparous female camels that have been subjected to difficult assisted births and that one stage repair of perineal lacerations improves the perineal conformation and helps to regain the normal fertility.
